# Naringenin promotes cell autophagy to improve high-fat-diet-induced atherosclerosis in ApoE^-/-^ mice

**DOI:** 10.1590/1414-431X20209764

**Published:** 2021-02-12

**Authors:** Ruifeng Zhao, Hanyan Xiao, Tao Jin, Feng Xu, Yan Li, Haiyan Li, Zhouyi Zhang, Yan Zhang

**Affiliations:** 1 Department of Interventional Therapy, Second Affiliated Hospital of Mudanjiang Medical University, Mudanjiang, Heilongjiang, China; 2 Department of Neurology, Second Affiliated Hospital of Mudanjiang Medical University, Mudanjiang, Heilongjiang, China; 3 Department of Geriatrics, Second Affiliated Hospital of Mudanjiang Medical University, Mudanjiang, Heilongjiang, China; 4 Department of Pain Management, Mudanjiang Medical University, Mudanjiang, Heilongjiang, China

**Keywords:** Atherosclerosis, Naringenin, Autophagy, Inflammation, High-fat, Apolipoprotein knockout mice

## Abstract

Naringenin (NAR) is a major flavanone in citrus fruits that has multiple pharmacological attributes such as anticancer and antiatherogenic. This study aims to investigate the mechanism of NAR in high-fat-diet (HFD)-induced atherosclerosis (AS) in apolipoprotein E-knockout (ApoE^-/-^) mice. A HFD-induced AS ApoE^-/-^ mouse model was established. The mice were treated with HFD, different doses of NAR and simvastatin (Simv). After drug treatment, the levels of total cholesterol (TC), triglyceride (TG), low density lipoprotein cholesterol (LDL-C), high density lipoprotein cholesterol (HDL-C), glutathione peroxidase (GSH-Px), malondialdehyde (MDA), superoxide dismutase (SOD), and alanine aminotransferase (ALT) were determined. The expression of interleukin-6 (IL-6) and tumor necrosis factor-α (TNF-α) was detected using qRT-PCR and enzyme-linked immunosorbent assay. The plaque area of the aorta of AS mice was determined using oil red O staining. Western blot analysis was applied to measure the levels of autophagy-related proteins [protein 1 light chain 3B (LC3B), beclin 1, and p62]. The TC, TG, LDL-C, TNF-α, ALT, and MDA levels were significantly increased while the HDL-C, SOD, and GSH-Px levels were decreased in the HFD-induced AS ApoE^-/-^ mice. NAR treatment reversed the expression of the above indicators in mice. After they were treated with different doses of NAR, the LC3B and beclin 1 levels were improved while the p62 protein level was decreased. This study suggested that NAR could promote cell autophagy to improve HFD-induced AS in ApoE^-/-^ mice.

## Introduction

Atherosclerosis (AS), a chronic disease of the arterial wall and a main cause of coronary artery disease, causes loss of productive life and death worldwide (1,2). Atherosclerotic lesions lead to stroke and myocardial infarction, which are responsible for cardiovascular diseases evolving as a major cause of death in industrialized countries (3). In recent years, AS has been delineated as a multifactorial disease since it may result from a combination of several factors such as male gender, hyperlipidemia, hypertension, and family history (4). Dysfunctions of endothelium in lesion-prone areas and of arterial vasculature are the main causes of atherosclerotic cardiovascular disease and the progress of AS (5,6). It is recognized that vascular chronic inflammation plays a crucial role in the initiation and progression of atherosclerotic lesions, from the appearance of the fatty streak to fat accumulation in acute coronary syndromes (7,8). There is an increasing awareness that cell autophagy may also be involved in the formation of AS plaques (9), but the potential mechanisms of cell autophagy in AS are still relatively unknown.

It is publicly recognized that a high intake of fruits and vegetables contributes to reducing cardiovascular disease risk due to the abundant flavonoids (10). Flavonoids, a family of polyphenolic compounds in vegetables and fruits, have been shown to possess anti-inflammatory functions (11). Naringenin (NAR) is a recognized member of bioflavonoids in citrus fruits, such as oranges, grapes, and berries, and the most important characteristic of NAR is its antioxidant capacity, which helps to reduce the burden of oxidative stress by reducing the production of free radicals (12). NAR is reported to have therapeutic potential in cardiovascular disorders and metabolic syndrome (13). NAR plays a cardiovascular protective role in umbilical vein endothelial cell injury by increasing autophagy flux (14). Importantly, the antiatherogenic effects of NAR have been reported in hypercholesterolemic rabbits and mice (10); however, relatively little is known about the related mechanisms. Taken together, this study was carried out to further investigate the mechanism of NAR in HFD-induced AS mice with the involvement of cell autophagy.

## Material and Methods

### Ethics statement

This study was approved and supervised by the Ethics Committee of the Second Affiliated Hospital of Mudanjiang Medical University (China). Significant efforts were made to minimize both the number of animals used as well as their suffering.

### Animal grouping and AS model establishment

A total of 48 specific-pathogen-free (SPF) male apolipoprotein E-knockout (ApoE^-/-^) mice (aged 4 weeks, weighing 20-22 g) (Beijing Vital River Laboratory Animal Technology Co., Ltd., China) were included and adaptively fed for 1 week. The high-fat-diet (HFD)-induced ApoE^-/-^ mice were randomly assigned to the HFD group [treated with high-fat diet (conventional feed + 21% fat + 0.15% cholesterol, provided by Beijing Keao Xieli Feed Co., Ltd., China)], the naringenin high (NAR-H) [treated with high-fat diet + high doses of NAR (100 mg/kg)], NAR middle (NAR-M) [treated with high-fat diet + middle doses of NAR (50 mg/kg)], NAR low (NAR-L) [treated with high-fat diet + low doses of NAR (25 mg/kg)] groups, and the simvastatin (Simv, No. 160409174, provided by China Meheco Topfond Pharma Co., Ltd., China) [treated with high-fat diet and Simv (10 mg/kg)] group, with 8 mice in each group. In addition, another 8 wild-type mice (C57BL/6J) were included and set as the control group. These treatments were conducted once a day. Mice in each group were fed for 12 weeks and had free access to food. Then, the mice were anesthetized and sacrificed, their blood was collected, and the aortas were dissected.

The mice in each group were fasted for 12 h after the last feed, and then they were intraperitoneally injected with 0.3 pentobarbital sodium (0.1 mL/10 g) (Solarbio Science & Technology Co., Ltd., China). The mice were then fixed on the fixation plate in a supine position and disinfected, and a longitudinal incision was produced at the right side of the midline neck. The tissues were cut open in layers, after which the right carotid artery was separated and its proximal end was clamped using a vessel clamp, and a rupture was produced in its distal end. Next, an arterial catheter containing 1% heparin saline was inserted through the rupture and the vessel clamp was released. The physiological condition of mice was observed by measuring the blood pressure using a multi-channel physiological signal acquisition system.

### Serum determination

Afterwards, the artery catheter was removed, and the carotid blood was collected after the right carotid artery was snipped. After 30 min, the blood was centrifuged at 1000 *g* for 15 min at 4°C after which the serum was collected and preserved at -80°C. Three serum samples in each group were collected, and the triglyceride (TG), total cholesterol (TC), low density lipoprotein cholesterol (LDL-C), high density lipoprotein cholesterol (HDL-C), superoxide dismutase (SOD), malondialdehyde (MDA), glutathione peroxidase (GSH-Px), and glutamic-pyruvic transaminase (ALT) levels in serum were measured.

### Enzyme-linked immunosorbent assay (ELISA)

Forty-eight h after model establishment, the aortic tissues of mice in each group were collected and washed with pre-cooled phosphate-buffered saline (PBS, 0.01M, pH=7.4). Then, the remaining blood was discarded and the tissues were weighed, shredded, and ground in a glass homogenizer containing PBS (9 mL PBS for 1 g of samples). Next, the homogenate was centrifuged at 7000 *g* at 4°C for 10 min and the supernatant was collected. Afterwards, the levels of inflammatory factors interleukin-6 (IL-6) and tumor necrosis factor-α (TNF-α) in the supernatant were detected according to the instructions of an ELISA kit.

### Quantitative reverse transcription polymerase chain reaction (qRT-PCR)

Aortic tissues of 3 mice were collected from each group to prepare tissue homogenate. Total RNA was extracted using the Trizol (Invitrogen, USA) one-step method, with the high-quality RNA identified by ultraviolet analysis and formaldehyde denaturing gel electrophoresis. After that, 1.0 μg RNA was collected and reversely transcribed into cDNA using avian myeloblastosis virus (AMV) reverse transcriptase. SYBR Green method was applied in qPCR. PCR primers were designed and synthesized by Shanghai Sangon Biotech Co., Ltd (China) (Table 1), with β-actin acting as an internal reference. The PCR reaction system was as follows: 1.0 μL cDNA, 10 μL 2× SYBR Green mix, 0.5 μL forward primer (10 μM), 0.5 μL reverse primer (10 μM), and added with RNase-free water until it reached 20 μL. The PCR reaction condition was as follows: pre-denaturation at 94°C for 5 min, followed by 40 cycles of denaturation at 94°C for 40 s, annealing 40 s at 60°C, and extension 1 min at 72°C, and a final extension at 72°C for 10 min. The production was identified using agarose gel electrophoresis. Data was analyzed using 2^-ΔΔCt^ method in which 2^-ΔΔCt^ refers to the ratio of the target gene expression between the experimental group and the control group. The formula was as follows: ΔΔCt = [Ct (target gene) - Ct (control gene)]_experimental group_ - [Ct (target gene) - Ct (control gene)]_control group_.

**Table 1:** Primer sequences of qRT-PCR.

Genes	Sequences
IL-6	Forward: 5′-CCAC TTCACAAGTCGGAGGCTTA-3′
	Reverse: 5′-CCAGTTTGGTAGCATCCATCATTTC-3′
TNF-α	Forward: 5′-GTCACCAAATCAGCGTTA-3′
	Reverse: 5′-CCACCATCAAGGACTCAA-3′
β-actin	Forward: 5′-GGCATCACACTTTCTACAACG-3′
	Reverse: 5′-GGCAGGAACATTAAAGGTTTC-3′

qRT-PCR: quantitative reverse transcription-polymerase chain reaction; IL-6: interleukin-6; TNF-α: tumor necrosis factor-α.

### Oil red O staining

Five mice from each group were anesthetized using diethyl ether and their chests were opened. After normal saline washing for 5 min through the ventricle, the aorta was dissected with the fibers and adipose tissues removed, and allowed to stand at room temperature for 15 min, followed by tap water washing twice (15 min each time) and distilled water washing for 2 min. The hearts were washed with 60% isopropanol and stained using oil red O (Beijing Solarbio Science & Technology Co., Ltd., China), and a photograph was taken using a high-resolution camera. AS was quantitatively analyzed by measuring the percentage of atherosclerotic plaque area to intimal area using PHOTOSHOP system (Adobe, USA).

### Western blot analysis

The proteins were extracted from the aortic tissues of mice using radio-immunoprecipitation assay (RIPA). After the protein concentration was determined by bicinchoninic acid (BCA) method, the proteins were added with loading buffer and denatured in a 100°C metal bath for 10 min. Then, 30 μg protein samples were treated with 10% sodium dodecyl sulfate-polyacrylamide gel electrophoresis (SDS-PAGE). After electrophoresis, the samples were transferred to a polyvinylidene fluoride (PVDF) membrane. Before incubation, the membranes were sealed with dried skimmed milk, which was dissolved in tris-buffered saline-Tween (TBST) buffer. After sealing, the membranes were cut according to the relative molecular mass of the protein, and incubated in a primary antibody diluted with 5% bovine serum albumin solution and a secondary antibody containing HRP (horseradish peroxidase). The membranes were rinsed with TBST after incubation, and an appropriate volume of HRP substrate was added to produce a band image in a fully automated optical imaging system. The image was further analyzed using Image Lab 4.0.1 (Bio-Rad Laboratories, USA) software.

### Statistical analysis

Differences between two groups were evaluated using the *t*-test while the differences among multiple groups were compared using one-way analysis of variance (ANOVA). The Statistical Package for the Social Sciences (SPSS) 17.0 (SPSS, Inc., USA) was applied for data analysis. P<0.05 was considered to be a statistically significant difference.

## Results

### NAR reduced the TG, TC, and LDC-C levels but enhanced the HDL-C level in serum in HFD-induced AS ApoE^-/-^ mice

Compared with the control group, the levels of TC, TG, and LDL-C in the HFD group were increased, while the HDL-C level was decreased (P<0.01), indicating that the ApoE^-/-^ mouse model was successfully established.

**Figure 1:**
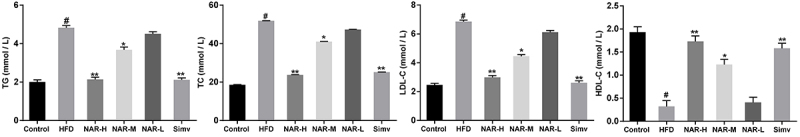
NAR reduced the TG, TC, and LDC-C levels but enhanced the HDL-C level in serum in HFD-induced atherosclerosis ApoE^-/-^ mice. Data are reported as mean±SD. ^#^P<0.01, *P<0.05 compared to the control group; **P<0.01, compared to the HFD group; n=8 (ANOVA). NAR: naringenin: high (H), medium (M), low (L); HFD: high-fat-diet; ApoE^-/-^: apolipoprotein E-knockout; Simv: simvastatin; TG: tri-glyceride; TC: total cholesterol; LDL-C: low density lipoprotein cholesterol; HDL-C: high density lipoprotein cholesterol.

After drug treatment of the HFD-induced AS ApoE^-/-^ mice, the levels of TG, TC, LDL-C, and HDL-C showed little difference between the NAR-L group and the HFD group (P>0.05). The NAR-H, NAR-M, and Simv groups showed decreased levels of TG, TC, and LDL-C, and an increased level of HDL-C (all P<0.01) (Figure 1).

**Figure 2:**
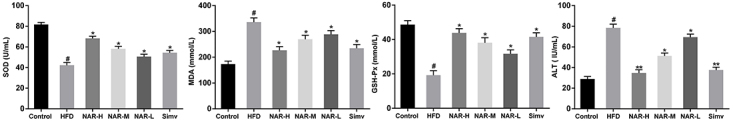
NAR decreased oxidative damage in HFD-induced atherosclerosis ApoE^-/-^ mice. Data are reported as mean±SD. ^#^P<0.01, *P<0.05, compared to the control group; **P<0.01, compared to the HFD group; n=8 (ANOVA). NAR: naringenin: high (H), medium (M), low (L); HFD: high-fat-diet; ApoE^-/-^: apolipoprotein E-knockout; Simv: simvastatin; SOD: superoxide dismutase; MDA: malondialdehyde; GSH-Px: glutathione peroxidase; ALT: alanine aminotransferase.

**Figure 3:**
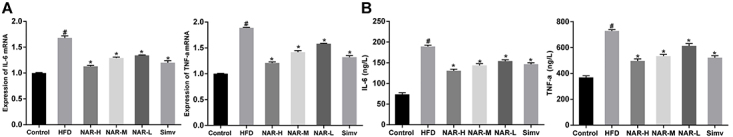
NAR decreased the inflammation in HFD-induced atherosclerosis ApoE^-/-^ mice. **A**, mRNA expression of IL-6 and TNF-α detected using qRT-PCR; **B**, protein levels of IL-6 and TNF-α. Data are reported as mean±SD. ^#^P<0.01, compared to the control group; *P<0.05, compared to the HFD group; n=8 (ANOVA). NAR: naringenin: high (H), medium (M), low (L) detected with ELISA; HFD: high-fat-diet; ApoE^-/-^: apolipoprotein E-knockout; Simv: simvastatin; IL-6: interleukin-6; TNF-α: tumor necrosis factor-α.

**Figure 4:**
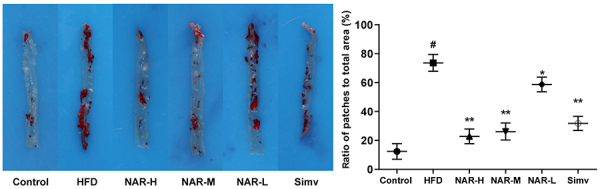
Photograph and graph showing that NAR reduced the plaque area in HFD-induced atherosclerosis ApoE^-/-^ mice. Data are reported as mean±SD. ^#^P<0.01, *P<0.05, compared to the control group; **P<0.01, compared to the HFD group; n=5 (ANOVA). NAR: naringenin: high (H), medium (M), low (L); HFD: high-fat-diet; ApoE^-/-^: apolipoprotein E-knockout; Simv: simvastatin.

**Figure 5:**
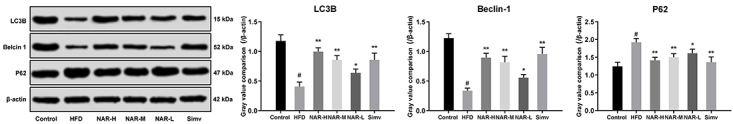
NAR enhanced expression of autophagy-related proteins in the aortic plaque in HFD-induced atherosclerosis ApoE^-/-^ mice. Protein expression of cell autophagy-related protein was detected using western blot analysis, n=3. Data are reported as mean±SD. ^#^P<0.01, *P<0.05, compared to the control group; **P<0.01, compared to the HFD group; n=5 (ANOVA). NAR: naringenin: high (H), medium (M), low (L); HFD: high-fat-diet; ApoE^-/-^: apolipoprotein E-knockout; Simv: simvastatin; LC3B: protein 1 light chain 3B.

### NAR decreased oxidative damage in HFD-induced AS ApoE^-/-^ mice

The detection of oxidative damage-related enzymes in HFD-induced AS ApoE^-/-^ mice demonstrated that the SOD and GSH-Px levels were lower while the ALT and MDA levels were higher in the HFD group compared with the control group. Compared with the HFD group, the SOD and GSH-Px levels in the NAR-L, NAR-M, NAR-H, and Simv groups were increased, while the ALT and MDA levels were decreased (all P<0.01) (Figure 2).

### NAR reduced inflammation in HFD-induced AS ApoE^-/-^ mice

The IL-6 and TNF-α mRNA expression was elevated in the HFD group compared with that in the control group, while the mRNA expression of IL-6 and TNF-α was decreased in the NAR-L, NAR-M, NAR-H, and Simv groups compared with those in the HFD group (all P<0.05) (Figure 3A).

The levels of IL-6 and TNF-α were enhanced in the HFD group compared with the control group, while these levels in the NAR-L, NAR-M, NAR-H, and Simv groups were lower than those in the HFD group (all P<0.05) (Figure 3B).

### NAR decreased the plaque area of the aorta in ApoE^-/-^ mice

The plaque area of the aorta in the HFD group was much larger than that of the control group. The plaque area was smaller in the NAR-L, NAR-M, NAR-H, and Simv groups compared with that of the HFD group, and importantly, the plaque area in the NAR-H group was smaller than that in the NAR-L and NAR-M groups (Figure 4).

### NAR increased levels of autophagy-related proteins in the aortic plaque in HFD-induced AS ApoE^-/-^ mice

Compared with the control group, the LC3B and beclin 1 protein levels were decreased in the HFD group (all P<0.05). Compared with the HFD group, the LC3B and beclin 1 protein levels were increased, while the p62 protein level was decreased in the NAR-L, NAR-M, NAR-H, and Simv groups (all P<0.05). These results indicated that NAR promoted the levels of autophagy-related proteins of the aortic plaque in HFD-induced AS ApoE^-/-^ mice (Figure 5B).

## Discussion

A key event in AS is a maladaptive inflammatory response to subendothelial lipoproteins resulting from failure in inflammation resolution, which promotes the transformation of atherosclerotic lesions into serious plaques (15). NAR has been reported to bear antiatherogenic and anti-cancer functions (16).

Importantly, our study demonstrated that the TG, TC, and LDL-C levels were higher while the HDL-C level was lower in HFD-induced AS ApoE^-/-^ mice. After different doses of NAR, the levels of these indicators were inversed in the NAR-H and NAR-M groups. Elevated TC and LDL-C levels lead to AS development (17). Increased level of LDL-C in serum is widely recognized as a risk factor in the promotion of endothelial dysfunction, which is also an important event in the progression of AS (10). Dyslipidemia in AS is characterized by increased TG and LDL-C, and decreased HDL-C (3). Low level of HDL-C is demonstrated to be correlated with enhanced risk of cardiovascular disease, and NAR and stalin combination treatment leads to reduced LDL-C and TG levels and improved HDL-C levels (18).

Oxidative damage in HFD-induced ApoE^-/-^ mice was reduced after they were treated with different doses of NAR, with enhanced SOD and GSH-Px levels and extremely decreased ALT and MDA levels. Oxidative stress caused by a disturbance of the equilibrium of reactive oxygen radicals and antioxidants, including excessive reactive oxygen species production and reduction of antioxidant capacity has been involved in the development of multiple cardiovascular diseases including AS (19,20). SOD and GSH-Px are two well-known antioxidant enzymes, which convert the highly-reactive radicals into less reactive ones to prevent water and molecular oxygen formation, or to form conjugates with some harmful compounds, thus producing safe rather than toxic substances (21,22). MDA is a main biomarker of oxidative stress (23), and MDA epitopes constitute the key mediators of atherosclerotic inflammation (24). NAR has been identified to show antioxidant activity and have a protective effect against lead-induced oxidative injury in mouse liver and kidney (25). Taken together, NAR could reduce the oxidative damage and improve the atherosclerotic lesions.

In addition, it has been demonstrated that inflammation drives the formation, development, and rupture of atherosclerotic plaques where the inflammatory subset of monocytes or macrophages accumulate and produce proinflammatory factors (26). Atherosclerotic plaques usually occurring at sites of mild coronary-artery stenosis can contribute to acute coronary syndromes (27). In the current study, we found that the area of plaques in the mouse aorta was reduced after NAR treatment. Furthermore, the levels of IL-6 and TNF-α were decreased. Increased levels of inflammation mediators, including IL-6 and TNF-α, are also main characteristics of AS (28). Previous study has shown that NAR suppresses the production of proinflammatory regulators IL-6 and TNF-α in lipopolysaccharide-stimulated mouse macrophages (29).

We further investigated cell autophagy in the atherosclerotic plaques in mice and found that the levels of autophagy-related proteins LC3B and beclin 1 were increased while the p62 level was decreased after NAR treatment. During autophagy, LC3B is transited to the adventitia of autophagosomes and therefore widely applied as an important marker of autophagosomal activity to evaluate autophagy or mitophagy (30). In autophagy, LC3-I converts into LCB-II, which can bind to p62, thus LC3B has been proven to be responsible for specific degradation of p62 via autophagy (31–33). A normal level of autophagy can improve AS by protecting plaque cells from oxidative stress and promoting cellular recovery (34). The up-regulation of autophagy and the balance between apoptosis and autophagy have been demonstrated to be effective in treating AS (35). 6-C-(E-phenylethenyl) NAR inhibits cell growth and induces cell cytoprotective autophagy in colon cancer cells (36). Silibinin, another type of flavonoid, has been shown to induce autophagy in human breast cancer cells (37). Taken together, our results showed that NAR enhanced the levels of LC3B and beclin 1 and reduced the p62 level, then induced cell autophagy in atherosclerotic plaque and reduced the inflammation of AS.

In summary, our study demonstrated that NAR had a positive effect on the atherosclerotic lesions. NAR treatment reduced the AS inflammation and oxidative damage, and then further reduced the atherosclerotic lesion in HFD-induced ApoE^-/-^ mice. Most of all, NAR induced cell autophagy in the atherosclerotic plaques. Hopefully, this study provides more evidence for the use of NAR in clinical treatment as an anti-inflammation agent. More studies will be carried out to explore the underlying mechanisms of the antiatherogenic function of NAR.
